# Extrapulmonary Tuberculosis Presenting As Stage V Bilateral Cervical Lymphadenitis With Cortical Cerebral Watershed Infarct Along With Maxillary and Sphenoid Sinusitis

**DOI:** 10.7759/cureus.56055

**Published:** 2024-03-12

**Authors:** Sankalp Yadav

**Affiliations:** 1 Medicine, Shri Madan Lal Khurana Chest Clinic, New Delhi, IND

**Keywords:** maxillary sinusitis, sphenoid sinusitis, watershed infarct, scrofula, lymphadenitis, extrapulmonary, antituberculous chemotherapy, tuberculosis, cervical lymphadenitis

## Abstract

Extrapulmonary tuberculosis is an infrequently reported condition. However, in endemic settings, it contributes to a significant number of cases. The most common site of extrapulmonary tuberculosis is the lymph nodes. Herein, an exceedingly rare case of extrapulmonary tuberculosis presenting as bilateral cervical lymphadenitis with external cerebral watershed infarct along with sphenoid and maxillary sinusitis in an Indian male is presented. A detailed literature search revealed that a case with all these clinical conditions together has never been reported to date. A diagnostic workup supported by radiometric investigations helped in the diagnosis, and timely management was initiated.

## Introduction

Tuberculosis remains a significant health challenge in developing nations, carrying vast social and economic consequences. Even in developed countries where the disease was once well controlled, it has resurfaced as a fresh health concern. This resurgence can be attributed to the migration of individuals from regions with high tuberculosis prevalence and the rising incidence of HIV infection in these areas [1].

Tuberculosis at sites other than the lungs is known as extrapulmonary tuberculosis. It constitutes about 8.4-13.7% of the total tuberculosis burden [2]. Tuberculous lymphadenitis is the most frequently encountered type of extrapulmonary tuberculosis. Among these cases, cervical lymph nodes are the most commonly affected, a condition often referred to as "scrofula" [1].

At the intersection of two major cerebral artery areas, there are watershed cerebral infarctions, also known as border zone infarcts. In these regions, the tissues are situated far from the direct arterial supply, rendering them susceptible to diminished perfusion. The decrease in perfusion in the distal areas of the arterial territories heightens their vulnerability to infarction [3]. Watershed cerebral infarctions make up approximately 5-10% of all cerebral infarctions [4]. They typically occur in older individuals, who have a higher prevalence of arterial stenosis, hypotensive episodes, and microemboli [5].

Because of its physical closeness to the nasal cavity and its relationship to the upper jaw's teeth, the maxillary sinus is the largest and most clinically significant air sinus and is primarily linked to rhinosinusitis [6]. However, sphenoid sinusitis is a rare entity and, if left untreated, can result in complications [7].

Herein, the first of its type cases of extrapulmonary tuberculosis presenting as bilateral cervical lymphadenitis with external cerebral watershed infarct with sphenoid and maxillary sinusitis in an Indian male is reported.

## Case presentation

A 42-year-old non-diabetic Indian male presented with a 1.5-month history of asthenia, purulent discharge from the right nostril, and bilateral cervical swelling. He also complained of pain on the right side of the face and intermittent episodes of left-sided throbbing headaches with the right nasal block for two weeks. Initially, the cervical swelling was unilateral on the right side, but during the course of one month, it ruptured. Subsequently, multiple swellings developed on the left cervical region (a few ruptured with purulent discharge). He had no fever, loss of appetite, cough, nausea, seizure, or loss of weight. He visited local clinicians for his pain management, who prescribed local analgesics for temporary relief.

He was a shoe factory worker by profession from a low socioeconomic background with no indicators of high-risk behavior. There was no history of trauma or tuberculosis in the close contacts. Further, there was no major medical or surgical intervention in the past.

A general examination revealed a male with these vitals: a temperature of 98.4 degrees Fahrenheit, a respiratory rate of 16/minute, a blood pressure of 130/80 mmHg, and a saturation of peripheral oxygen of 99% on room air. There was no icterus, pallor, cyanosis, edema, clubbing, or koilonychia present.

**Figure 1:**
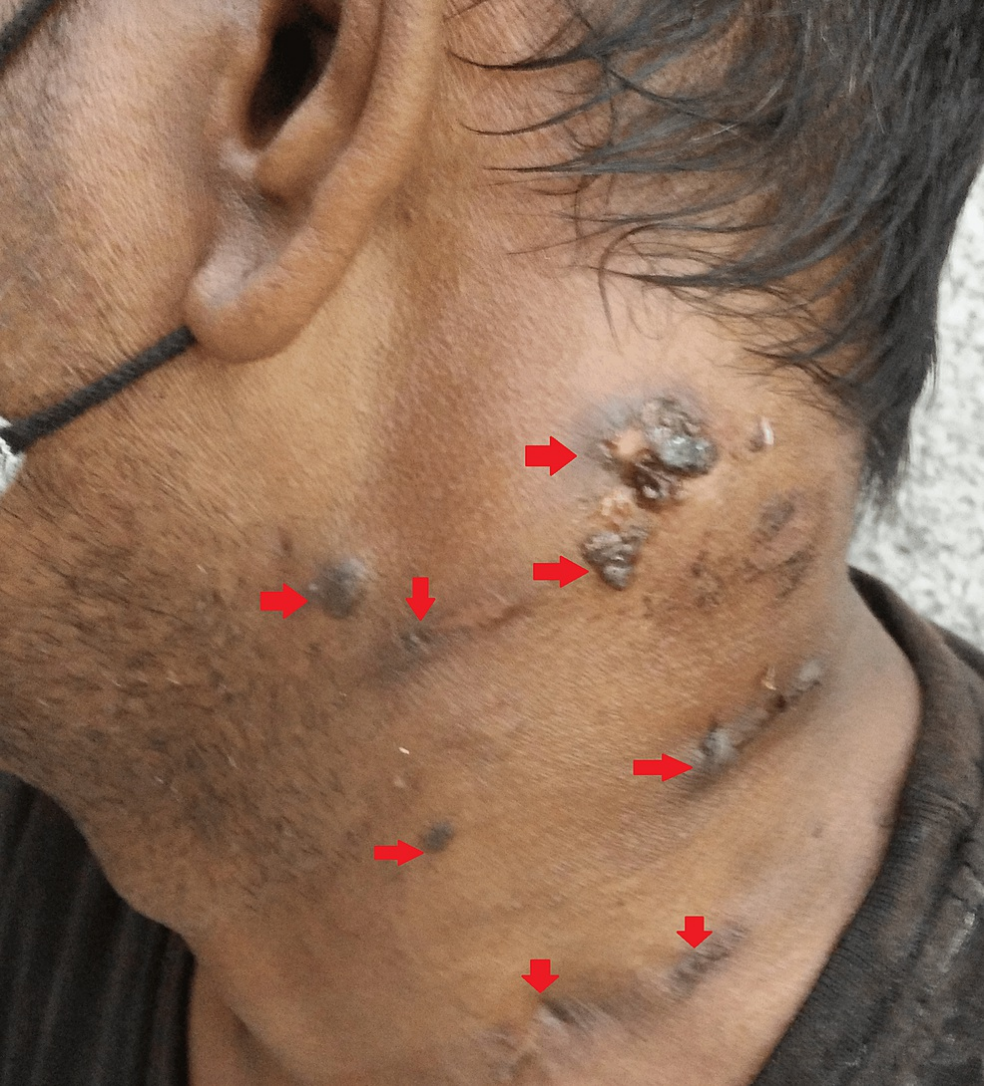
Gross image of the left side at the presentation

**Figure 2:**
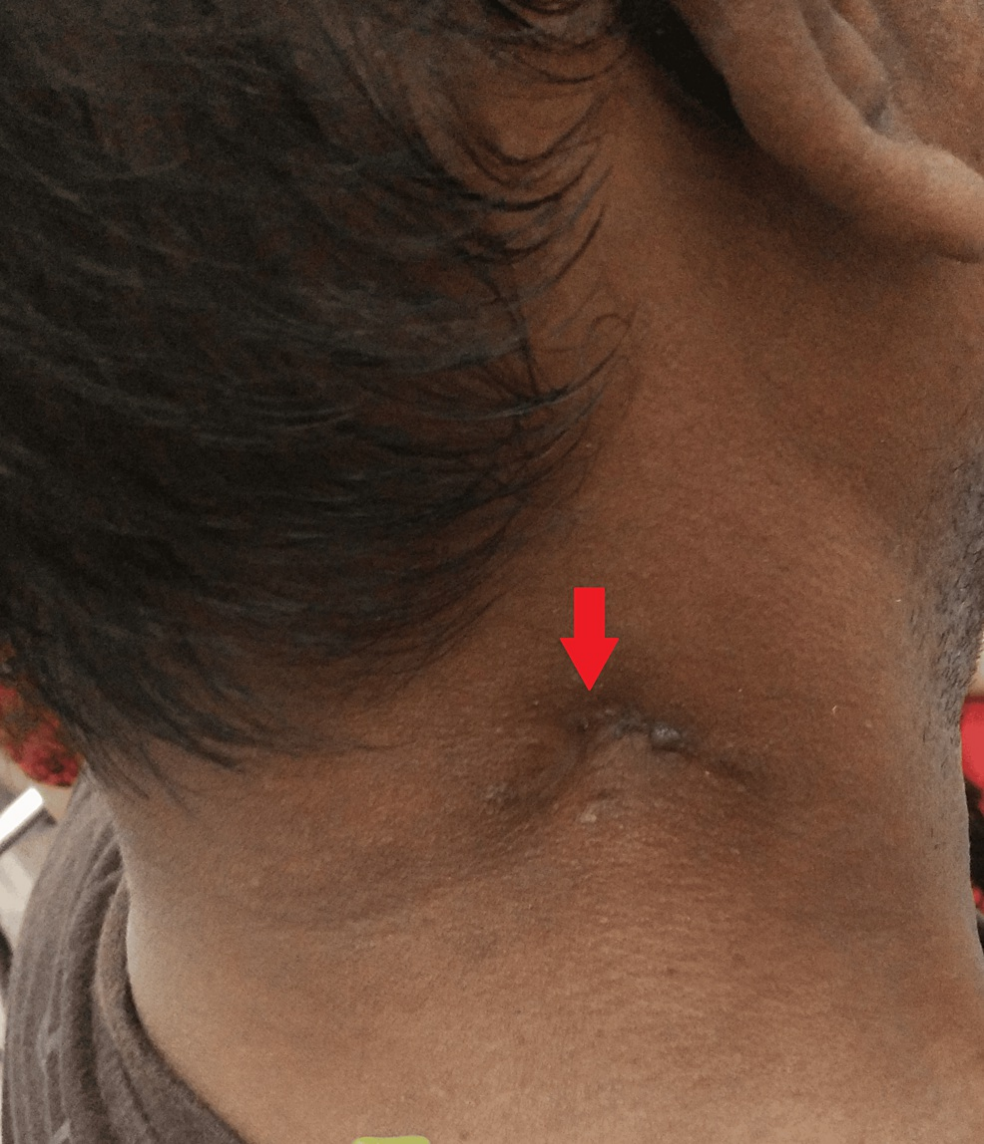
Gross image of the right side at the presentation

Upon local examination, one healed scar was observed in the right posterior cervical region. On the left side, various swellings were noted: the largest measured 3 cm x 2 cm with a discharging sinus at level Va; another, approximately 1 cm x 1 cm in size, exhibited a firm consistency, was painless, and lacked a discharging sinus, located just below the former; two swellings were ulcerated with two discharging sinuses at level Vb; a swelling measuring 1 cm x 1 cm was present at level III; two swellings with a healed scar and a discharging sinus were observed at level IV; additionally, one swelling, approximately 1 cm x 0.5 cm in size, was firm in consistency, painless, and lacked a discharging sinus, located just above those at level III; finally, a healed scar was noted at level III (Figures 1-2).

His examination of the ear, nose, and throat revealed tenderness on palpation at the right side of the face around the maxillary sinus. However, there was no deviation of the nasal septum or nasal polyps but a purulent, non-foul-smelling discharge from the right nostrils.

His systemic examination revealed normal respiratory, cardiovascular, and abdominal systems. A detailed examination of the central nervous system showed that the intellectual functions were within the normal range, and all cranial nerves exhibited normal function. Besides, there were no engorged veins. Upon examination of the motor system, the bilateral upper and lower limbs displayed normal characteristics. However, there was hypotonia in the left upper limb. Superficial reflexes were normal, and corneal and conjunctival responses were unremarkable. Abdominal reflexes and deep tendon reflexes were normal. Sensory assessment revealed normal responses to pain, temperature, pressure, vibration, and joint sensations in all limbs. No cerebellar signs were noted on either side. There were no indications of meningeal irritation, and examinations of the skull and spine yielded normal findings. The gait assessment was not suggestive of any weakness or abnormality.

An extensive laboratory workup was done, as mentioned in Table 1.

**Table 1:** Laboratory workup AFB: Acid-fast bacilli; CBNAAT: Cartridge-based nucleic acid amplification test; Mtb: *Mycobacterium tuberculosis;* CSF: Cerebrospinal fluid

Test	Result	Reference range
Hemoglobin	10.3 g/dL	11.9-15
Platelet count	1.8 x 10^9^/L	1.5-4.0 x 10^9^
Total leukocyte count	5.4 × 10^9^/L	4-10
Erythrocyte sedimentation rate	53 mm/hr	0-20
Bilirubin (conjugated)	0.8 µmol/L	<1
Human immunodeficiency virus	Non-reactive	
Fasting blood sugar	4.20 mmol/L	3.9-5.6
Serum sodium	137	135-145 mEq/L
Serum potassium	4	3.5 to 5.5 mEq/L
Serum magnesium	1.9	1.6–2.5 mg/dl
Serum calcium	4.9	4.3 to 5.3 mEq/L
Serum creatinine	54 µmol/L	53-97.2
Serum thyroid-stimulating hormone levels	0.6 mU/L	0.4-4.0
Serum uric acid	4.8	3.5-7.2 mg/dL
Electrocardiogram	Normal	
Sputum for AFB	Negative	
CBNAAT of sputum	Negative for Mtb	
Culture of nasal discharge from right nostril	Positive for Mtb	
Microscopy with potassium hydroxide	Negative	
CBNAAT of the pus from bilateral cervical lymph nodes	Positive for Mtb with no resistance to rifampicin	
Histopathology of the bilateral cervical lymph nodes	Suggestive of necrosis with a few Langhans giant cells and scattered lymphocytes with AFB	
Culture of pus from the bilateral cervical lymph nodes	Growth of Mtb seen	
Hepatitis panel (A, B, and C)	Negative	
CSF examination	Normal	
C-reactive protein	5 mg/dL	0.3 to 1.0 mg/dL

**Figure 3:**
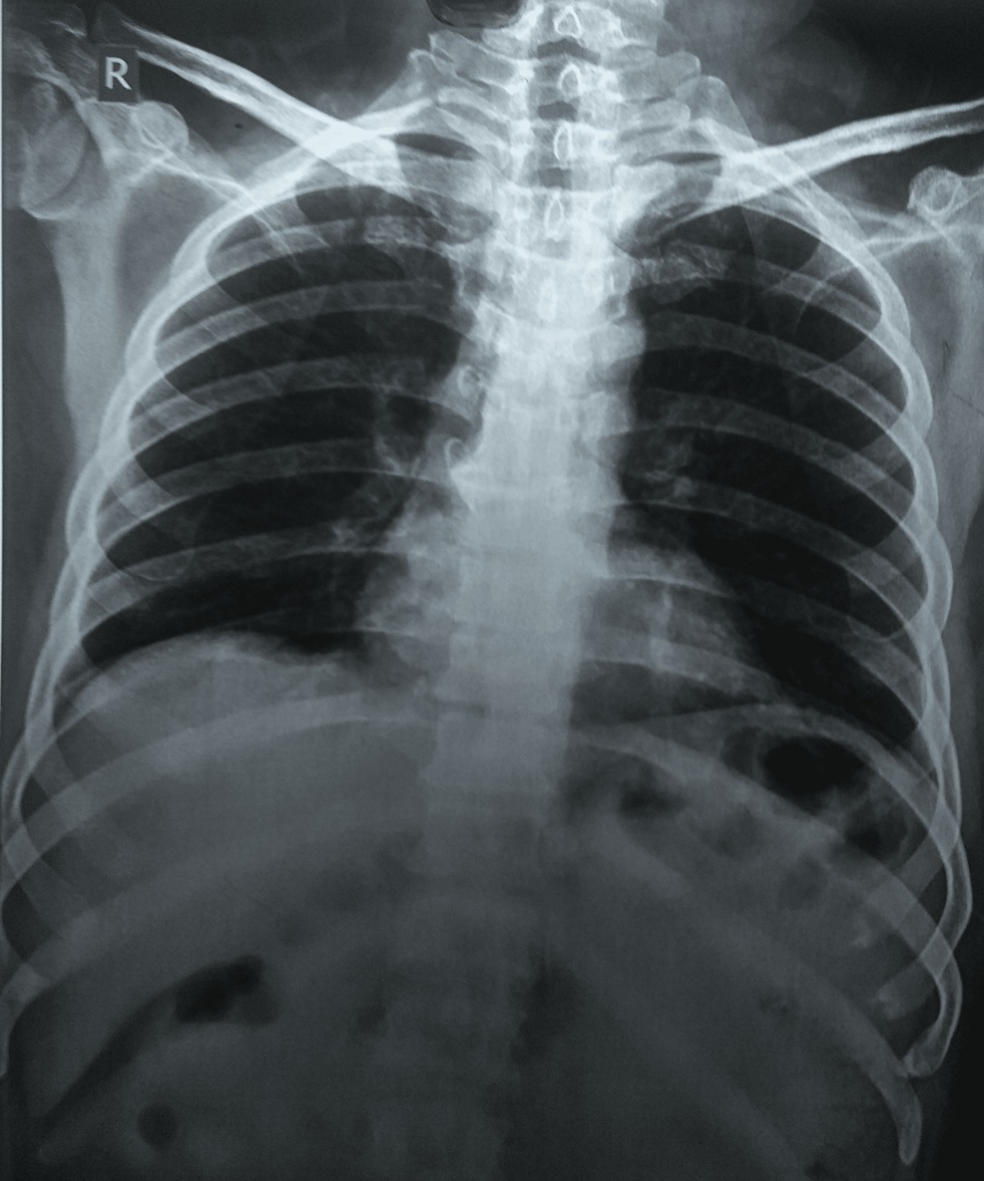
A normal chest radiograph

A chest radiograph was normal (Figure 3).

**Figure 4:**
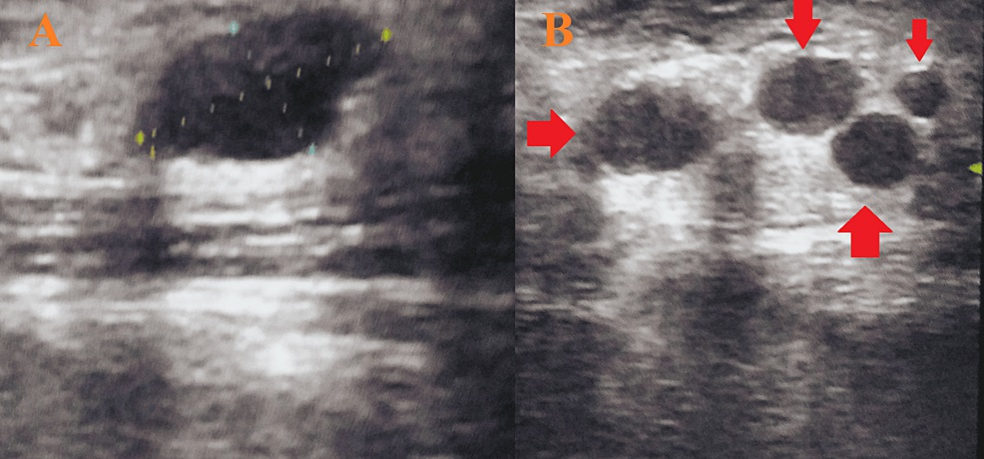
Ultrasound of the neck suggestive of multiple deep cervical lymphadenopathies on the left side A: The largest lymph node on the right side; B: Multiple deep cervical lymphadenopathies on the left side

An ultrasound of the neck was suggestive of multiple bilateral deep cervical lymphadenopathies, with the largest measuring 16 x 6 mm on the right and 26 x 14 mm on the left side (Figure 4).

**Figure 5:**
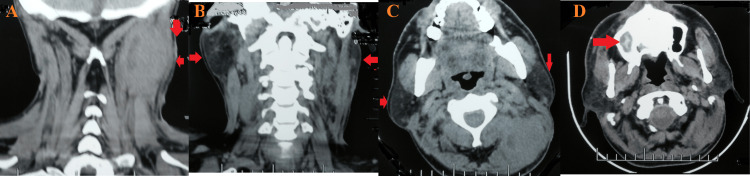
A plain CT of the neck CT: Computed tomography A: Left cervical lymph node involvement B: Bilateral cervical lymph node involvement C: Bilateral cervical lymph node involvement D: Right maxillary sinus involvement

A plain computed tomography of the neck showed multiple cervical lymph nodes on the left side at levels II, III, IV, and V, the largest measuring 1.7 x 1.2 cm. Degenerative changes were seen in the cervical spine. Diffuse mucosal thickening with calcification was seen within the right maxillary sinus, with mild mucosal thickening on the left sphenoid sinus (Figure 5).

**Figure 6:**
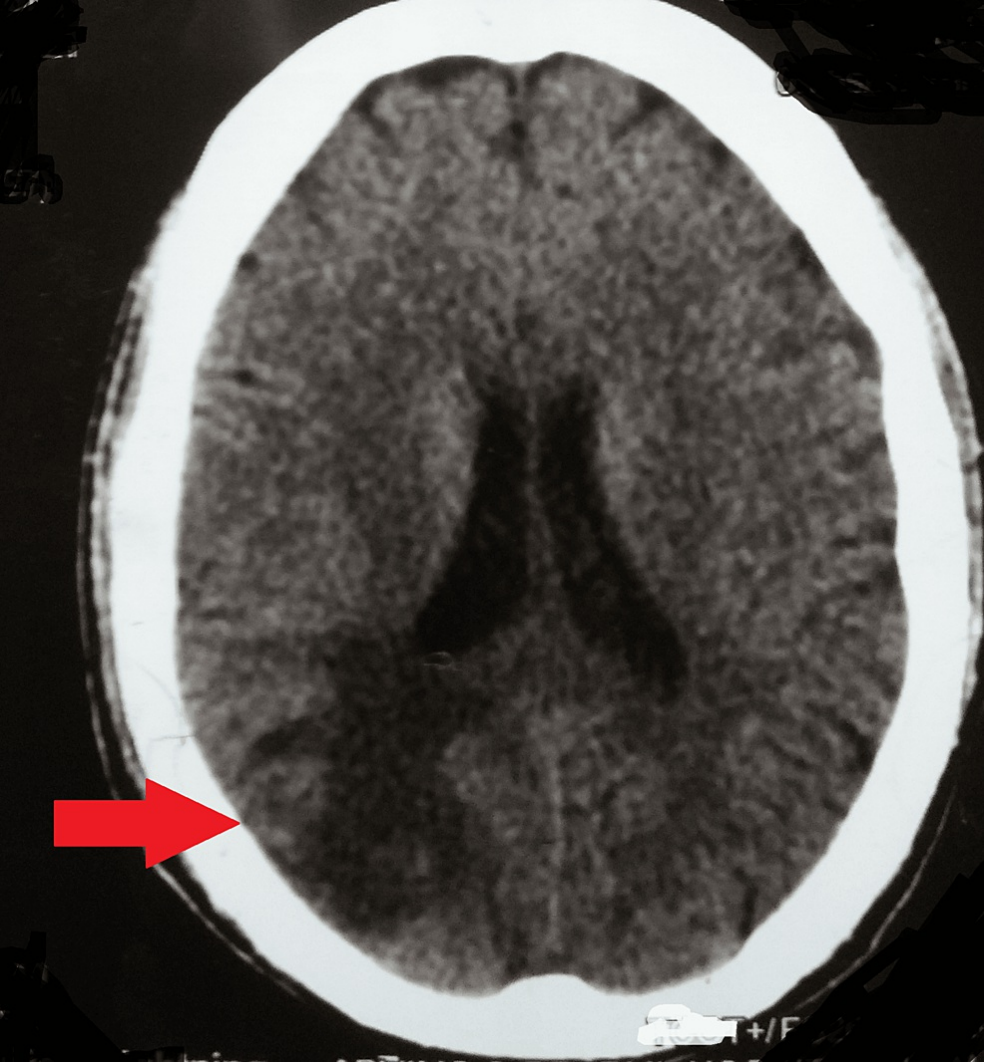
An MRI of the brain showed involvement of the right cerebral hemisphere with border zone infarct MRI: Magnetic resonance imaging

Magnetic resonance imaging of the brain showed involvement of the right cerebral hemisphere with border zone infarcts or an external watershed infarct in the occipital cortex between the middle and posterior cerebral arteries (Figure 6).

Magnetic resonance angiography of the intracranial arteries revealed irregular, mild constriction of the right middle cerebral artery's M2 segment and scarcity of cortical branches of the bilateral middle cerebral arteries, as well as severe narrowing of the right posterior cerebral artery's P1 segment with blockage of the petrous segment.

An antigravity aspiration of pus was attempted, but it was not successful, but a repeat fine needle aspiration biopsy yielded 5 ml of thick pus from the left side and 2 ml from the right side. Samples were sent for histopathology and cartridge-based nucleic acid amplification test (Table 1).

Based on the investigations, a definite diagnosis of tuberculous stage V bilateral cervical lymphadenitis with cortical cerebral watershed infarct and maxillary and sphenoid sinusitis was made, and he was put on treatment with a fixed-dose combination of antituberculous drugs (rifampicin, isoniazid, ethambutol, and pyrazinamide) along with antiplatelet therapy, statins, and topical nasal decongestants. Per the national policy, he was offered incision and drainage of the infected lymph nodes, but he was unwilling to do so. Additionally, he was counseled to maintain hygiene and treatment adherence. He continued his treatment, and the same was suggested by marked improvements at the third-month follow-up, where there was remarkable wound healing with scar formations.

Furthermore, there was a decrease in pain experienced over his right maxillary sinus. He completed his six months of treatment, and the ultrasound of the neck was repeated, which showed a marked reduction in the size of lymph nodes.

**Figure 7:**
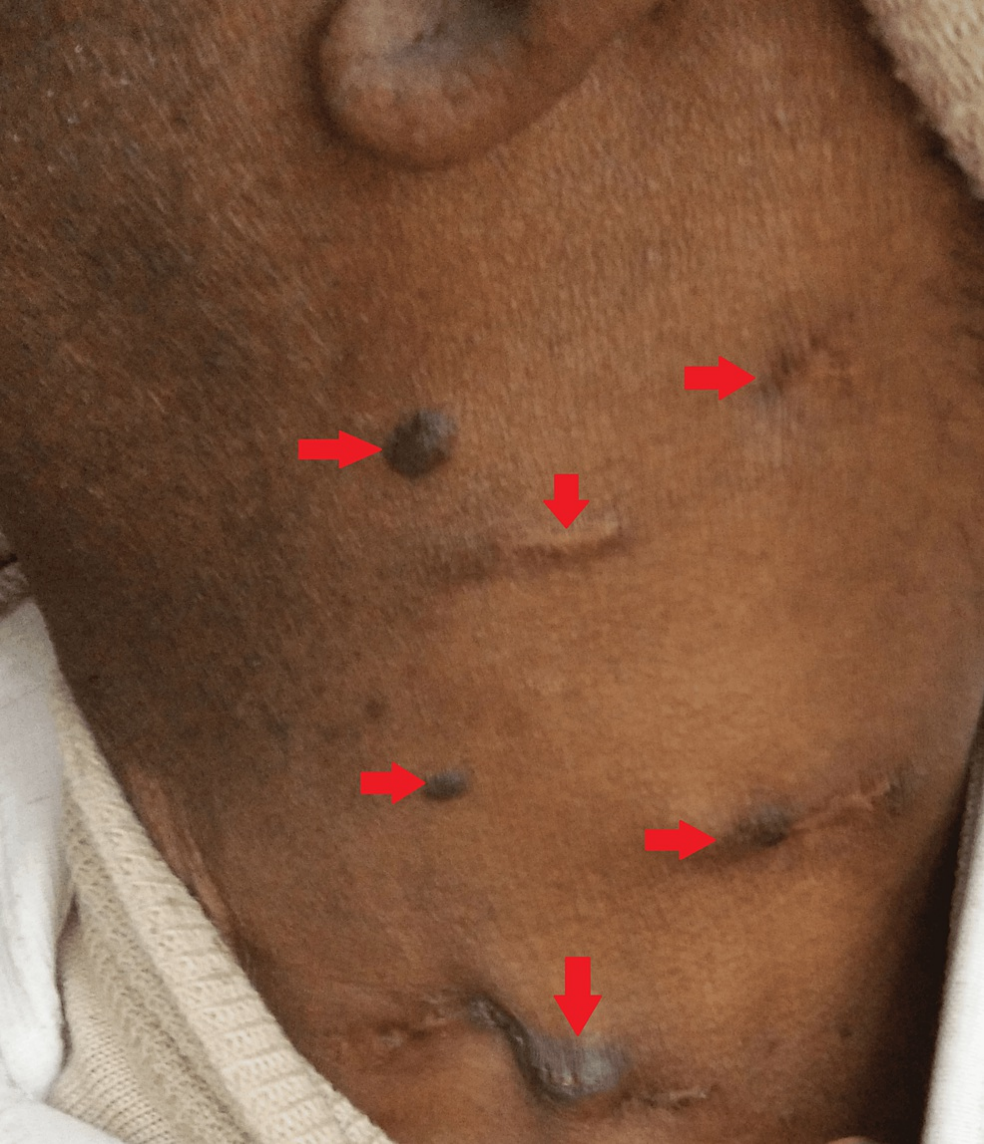
Gross image at the six-month follow-up of the left side

**Figure 8:**
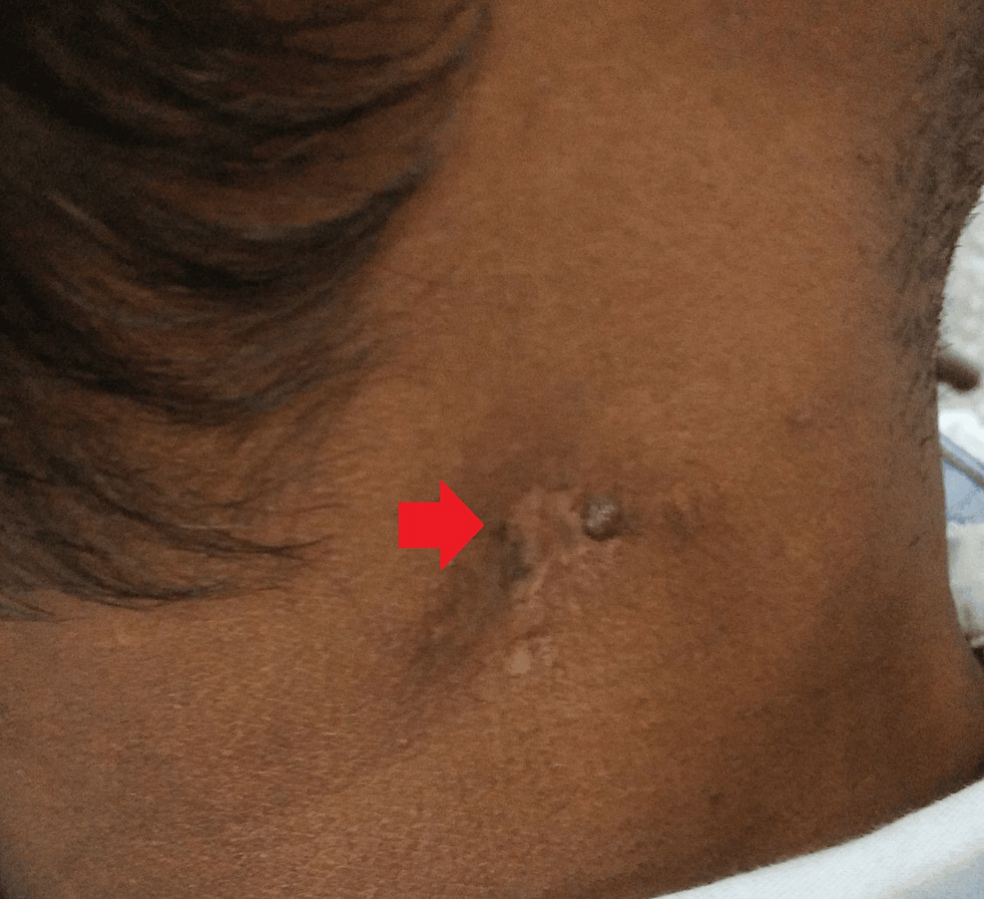
Gross image at the six-month follow-up of the right side

However, for complete resolution, his treatment was extended for the next three months per the national guidelines. The latest gross images show a near-complete resolution of the disease (Figures 7-8).

## Discussion

Tuberculosis, dubbed the "Captain of all these men of death" by John Bunyan in the 18th century, remains one of the most significant health challenges worldwide [1]. Peripheral lymph node tuberculosis stands as the prevailing type of extrapulmonary tuberculosis [8]. Cervical tuberculous lymphadenopathy, historically known as "scrofula," was once treated in medieval England with the "King’s touch" and the granting of a "gold coin." Today, it remains the leading cause of persistent cervical lymph node enlargement in developing countries like India [1].

This condition is typically attributed to *Mycobacterium tuberculosis*, although atypical mycobacteria such as *Mycobacterium avium,* and* Mycobacterium kansasii* have also been documented, particularly in cases involving children [9]. Interestingly, Jones and Campbell have delineated five stages characterizing the progression of tuberculous lymphadenitis. In the present case, the patient presented at stage 5, marked by the formation of sinus tracts (Table 2) [10].

**Table 2:** Jones and Campbell classification of tuberculous lymphadenitis Reference [10]

Stages of progression of tuberculous lymphadenitis
Stage 1: Enlarged, firm, mobile, discrete nodes showing non-specific reactive hyperplasia
Stage 2: Large rubbery nodes fixed to surrounding tissue owing to periadenitis
Stage 3: Central softening due to abscess formation
Stage 4: Collar-stud abscess formation
Stage 5: Sinus tract formation

Where two major cerebral artery areas converge is where watershed cerebral infarctions, also called border zone infarcts, occur. Tissues in these locations are not directly supplied by arteries, which puts them at risk for decreased blood flow. The distal portions of the artery areas are more vulnerable to infarction due to decreased perfusion [3,4]. There are two recognized forms of watershed cerebral infarctions: internal (subcortical) and exterior (cortical). Whereas external watershed cerebral infarctions are caused by embolism and sometimes occur in conjunction with hypoperfusion, internal watershed cerebral infarctions are the consequence of hypoperfusion. Watershed infarctions can be detected using sophisticated imaging methods such as transcranial doppler ultrasonography and diffusion-weighted magnetic resonance imaging [4]. According to the border zone infarct classification, this particular patient had an external watershed infarct in the occipital cortex between the middle and posterior cerebral arteries [5]. The treatment modalities for different types of watershed cerebral infarctions vary depending on their underlying causes [4].

While numerous risk factors for stroke have been identified, previous studies have suggested that primary infections, such as tuberculosis (with or without involvement of the central nervous system), may pose an additional risk of stroke. Lee et al., in their study over 3.8 years, reported that the overall risk of ischemic stroke was higher in tuberculosis patients compared with the matched non-tuberculosis cases [11]. The heightened immune response to tuberculosis infection observed in the patients plays a significant role in triggering platelet activation and fostering the development of a hypercoagulable state. These factors were implicated in the pathogenesis of pulmonary tuberculosis-related ischemic stroke [12].

Rhinosinusitis refers to inflammation of the nasal mucosa and paranasal sinuses. The sinus that is most frequently afflicted is the maxillary sinus. The removal of microorganisms and foreign objects from the maxillary sinus is largely dependent on the mucosa of the sinus [13]. Acute viral rhinitis and allergic rhinitis are closely related to sinusitis symptoms [6]. An upper respiratory tract viral infection or related allergies have been identified as a predisposing factor. Intrasinus calcification is observed in several conditions, including inflammatory disorders and benign or malignant tumors. It is frequently associated with fungal sinusitis, particularly aspergillosis, as previously documented. While intrasinus calcification can also manifest in nonfungal inflammatory diseases like mucocele or bacterial sinusitis, it is relatively rare in such cases. Limited literature exists regarding intrasinus calcification in nonfungal sinusitis [14].

The occurrence of paranasal sinus tuberculosis without involvement of the nasal cavity, as observed in the current case, is exceedingly rare [15]. Sinonasal tuberculosis typically occurs through direct transmission of infected droplets from patients with pulmonary tuberculosis or through bloodstream dissemination. It is most commonly observed in adults and manifests with symptoms such as nasal discharge, nasal stuffiness, epistaxis, and crusting of the nasal passages. Sinonasal tuberculosis presents three distinct types of pathological changes. The first type involves infection confined to the mucosal lining, potentially resulting in polyp formation and sinus obstruction accompanied by purulent discharge. In the second type, bone involvement occurs, leading to the formation of fistulous tracts and discharge containing tuberculosis bacilli. The third type is characterized by the development of tuberculomas due to mucosal hyperplasia and granuloma formation. Untreated sinonasal tuberculosis can progress to complications such as septal perforation, atrophic rhinitis, or nasal cavity stenosis [16].

Sphenoid sinusitis is a relatively rare infection, comprising about 3% of all cases of acute sinusitis [17]. It typically presents alongside pansinusitis, although there are rare instances where it occurs in isolation. Moreover, due to the proximity of the sphenoid sinus to several structures in the head, like the sixth cranial nerve, cavernous sinus, internal carotid artery, pituitary gland, sphenopalatine artery, adjacent dura mater, etc., complications can be fatal [7].

The diagnosis of these cases with the involvement of various sites, especially in the absence of pulmonary involvement, is challenging. Often times, cervical lymphadenitis is not associated with classical features of tuberculosis, and a wide variety of differentials make the task really difficult [9]. Management is usually conservative for cervical lymphadenitis and involves a course of antituberculous drugs. The national guidelines recommended by the World Health Organization recommend upfront drug susceptibility testing, as done in the present case, to rule out drug resistance [18].

In short, the first case of its type, i.e., extrapulmonary tuberculosis presenting as stage V bilateral cervical lymphadenitis with cortical cerebral watershed infarct and maxillary and sphenoid sinusitis, is detailed here. There is a limitation of only one case, but this case is exceedingly essential to disseminating knowledge about these clinical entities together.

## Conclusions

An immunocompetent Indian male with concomitant involvement of bilateral cervical lymph nodes with cortical cerebral watershed infarct and maxillary and sphenoid sinusitis is presented here. It is very important to create awareness about these conditions, which are never reported even in endemic countries. Prompt management is always essential to avoid any fatal outcomes.
